# Robotic liver surgery: a global snapshot. Results from an international survey

**DOI:** 10.1007/s00464-026-12582-2

**Published:** 2026-03-03

**Authors:** Silvio Caringi, Antonella Delvecchio, Annachiara Casella, Valentina Ferraro, Matteo Stasi, Nunzio Tralli, Tommaso Maria Manzia, Michele Tedeschi, Riccardo Memeo

**Affiliations:** 1 Unit of Hepato-Biliary and Pancreatic Surgery, “F. Miulli” General Hospital, Acquaviva delle Fonti, 70021 Bari, Italy; 2 Department of Surgery, Università Degli Studi Roma “Tor Vergata”, Via Montpellier1, 00133 Rome, Italy; 3 Department of Medicine and Surgery, LUM University, 70010 Casamassima, Italy; 4 Department of Surgery Sciences, Transplant and HPB Unit, University of Rome Tor Vergata, Rome, Italy

**Keywords:** Robotic liver surgery, Learning curve, Surgical decision-making, Minimally invasive liver surgery, International survey

## Abstract

**Background:**

Robotic liver surgery has gradually increased within the realm of minimally invasive hepatobiliary surgery; nevertheless, worldwide adoption rates, educational systems, and thought processes are various.

**Materials and methods:**

An online worldwide survey was devised to collect data from hepatobiliary surgeons with experience or interests in robotic liver surgery. The design explores the use of robotic platforms, adoption rate, learning opportunities, learning curves, procedural options based on complexity, safety in perioperative phases, and limiting factors. Descriptive statistics are used to analyze the collected responses.

**Results:**

The da Vinci platform is the most commonly used, while the new systems are still in the early adoption stages. Variability of use patterns has been identified. Structured learning, including simulation, proctoring, and learning at a second console, has been identified as essential to ensure safe adoption. The learning curve is a multi-step process that is dependent on procedure type, inherent surgical skills, and prior training. The laparoscopic, robotic, and open methods are considered to be relatively similar in low-complexity resections, while robotic, open, or a combination of robotic or open would be preferred for directing posterosuperior, major hepatectomy, as well as reconstruction cases, respectively.

**Conclusion:**

Robotic liver surgery is gradually being adopted within the realm of hepatobiliary surgery, but has been unevenly distributed. Uniform models of training, organizational structure, and equal availability of systems are essential factors that define how such systems are expanded.

Minimally invasive liver surgery has rapidly evolved over the past two decades with technological innovation and expanding institutional expertise worldwide. Within this evolution, robotic-assisted liver surgery has emerged as a valuable complement to conventional laparoscopy, offering enhanced dexterity, stable three-dimensional visualization, and improved ergonomics that facilitate complex hepatic resections, including major and post-chemotherapy procedures [1–4]. Several contemporary series and multi-institutional studies have demonstrated that robotic liver resection (RLS) achieves perioperative outcomes comparable to laparoscopic and open approaches, with particular advantages in intracorporeal suturing, precise parenchymal dissection, and procedures involving posterior or superior segments [5–9].

Despite this growing body of evidence, global adoption of RLS remains inhomogeneous and is clearly influenced by issues of platform availability and cost, variability in surgical case volume, and a lack of standardised training pathways. Recent surveys and expert analyses have underlined the structured curricula, dual-console mentorship, and stepwise progression as perceived key elements in safe introduction and skills acquisition [10–13]. However, true determinants of the learning curve remain incompletely defined, with considerations also focusing on the interplay between prior open or laparoscopic experience and robotic proficiency.

In this context, international surveys represent a rare opportunity to capture real-life practice patterns and the perspective of surgeons. This study represents an updated global snapshot that exclusively focuses on robotic liver surgery, addressing platform diffusion, training pathways, perceived dynamics of learning curves, and approach selection at various levels of procedural complexity. This survey, therefore, tries to outline current trends and identify remaining barriers that may inform future training frameworks and resource allocation by incorporating the standpoint of international HPB surgeons, including contributions from centers with active robotic liver surgery programs.

## Materials and methods

This study was designed as a cross-sectional, international, web-based survey aimed at evaluating contemporary trends, patterns of adoption, training pathways, and perceived challenges associated with robotic HPB surgery. The survey was developed by a panel of HPB surgeons with expertise in minimally invasive and robotic surgery. The questionnaire included demographic items, institutional characteristics, surgical experience, exposure to minimally invasive HPB surgery, robotic platform availability, learning curve perception, training modalities, and perceived advantages and limitations of robotic HPB procedures.

The survey consisted of 36 structured questions, mostly multiple-choice with some open-ended fields, designed to ensure standardization and reduce response ambiguity.

The survey was disseminated internationally through multiple surgical societies, including the Italian Association of Hospital Surgeons (ACOI), the Italian Society of Endoscopic Surgery (SICE), the European Association for Endoscopic Surgery (EAES), and the International Hepato-Pancreato-Biliary Association (IHPBA). Dissemination strategies included institutional mailing lists, newsletters, and dedicated communication channels of each society. In addition, the survey link was shared on major social media platforms commonly used for professional medical networking (e.g., LinkedIn), a strategy that has been increasingly recognized as effective for reaching geographically diverse surgical communities.

A total of 242 responses were collected between June 2025 and November 2025.

To ensure that the analysis reflected the perspectives of surgeons actively involved in robotic HPB surgery, we applied predefined inclusion and exclusion criteria. Responses were excluded if:

1. The participant self-identified as a resident or trainee.
2. The participant reported no access to a robotic platform at their current institution.
3. Surgical experience was less than 5 years, consistent with the literature defining early career thresholds for HPB independence.
4. Responses were duplicate entries (same email or identifiable metadata).

After applying these criteria and removing incomplete or inconsistent entries, 119 responses were retained for final analysis.

Data were exported from Google Forms into Microsoft Excel and subsequently analyzed using Python (Pandas library). Descriptive statistics were used to summarize demographic characteristics and survey responses. Categorical variables were reported as frequencies and percentages.

Given the exploratory nature of the study and the descriptive intent of the survey, no inferential statistical testing was planned. This approach is consistent with methodological standards for international surgical surveys, where the aim is to capture practice patterns rather than test hypotheses.

Participation was voluntary, and consent was implied upon submission. No patient data were collected. According to international guidelines for online surveys and institutional policies, this study was exempt from formal ethical approval.

## Results

Respondents were predominantly (99; 83.2%) male, while 19 (16%) identified as female and 1 (0.8%) preferred not to disclose their gender. In terms of professional position, the cohort was mainly composed of consultants (64; 53.8%), followed by chiefs or heads of unit (41; 34.5%), fellows (8; 6.7%), and a smaller proportion reporting other roles (6; 5%). Surgical experience was largely concentrated among surgeons with more than 10 years of practice (84; 70.6%), with the remainder reporting 5–10 years of experience (35; 29.4%).

**Fig. 1:**
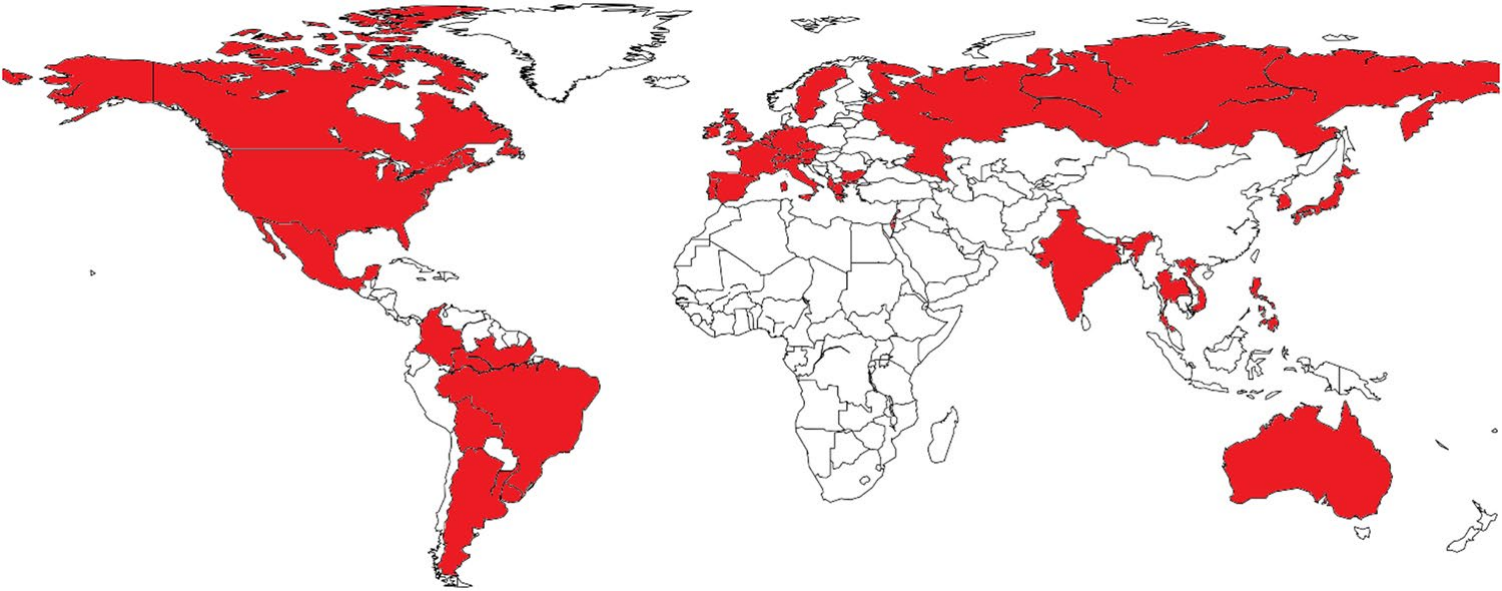
Countries of origin of survey participants (red) (Color figure online)

The survey demonstrated wide international participation, as shown in Fig. 1.

In Europe, the biggest contingents were from Italy (41; 34.5%), Spain (9; 7.6%), and France (6; 5.0%). The largest Asian contingents included those from India (8; 6.7%) and South Korea (5; 4.2%). The biggest part of the South American contribution came from Brazil, with 9 questionnaires (7.6%). The remaining answers came from other countries, each of them contributing less than 5% individually. This broad distribution reflects the global diffusion of robotic HPB surgery and supports the external validity of the survey.

Regarding access to minimally invasive technologies, all respondents indicated the availability of at least one robotic platform in their institution. The da Vinci system was the most frequently reported, with 112 (94.1%) respondents indicating exclusive use of this model. A smaller number of participants (6; 5%) mentioned alternative platforms such as Versius®, Hugo™ RAS, Toumai®, and Hinotori™. In addition, 3 (2.5%) participants indicated access to multi-platform configurations.

Respondents were asked how they had completed their robotic surgery learning curve, and various pathways were reported. The majority (77.3%) had progressed through a structured robotic curriculum, often with a mixed approach of proctorship, case observation, and simulator-based training. Only a minority reported having completed their learning curve without formal structured support. Most specifically, 64.7% of the respondents recognized that the dual-console system contributed meaningfully to facilitating the robotic learning curve by enhancing real-time mentoring, sharing visual fields, and allowing for the safe exchange of operative roles during training.

**Fig. 2:**
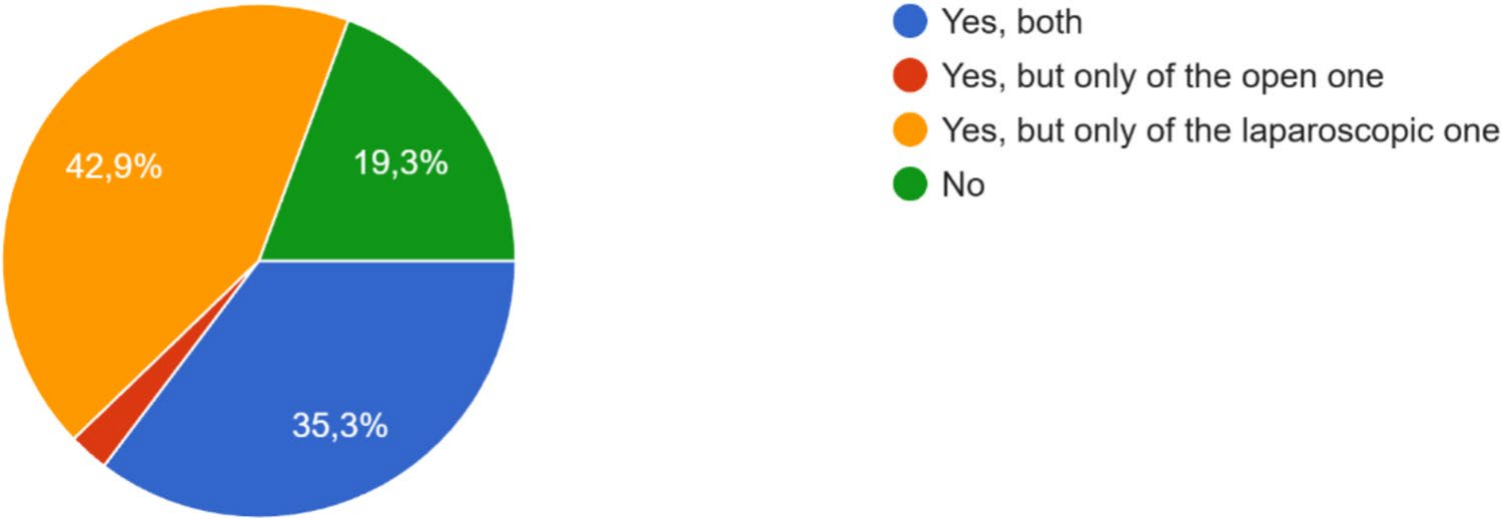
Responses to the survey question asking participants whether the learning curve of robotic surgery is easier and/or faster compared with open and laparoscopic liver surgery. Data are expressed as percentages of respondents for each response option

These findings notably parallel the answers from the preceding questions exploring learning curve completion in open and laparoscopic surgery, where respondents most commonly indicated that experience was gained primarily through direct operative exposure with progressive autonomy. When asked “Do you think that robotic learning curve is easier/faster than open/laparoscopic one?”, opinions were divided (Fig. 2). A great number of respondents (80.7%) claimed that robotics had a shorter and more intuitive learning curve because of improved visualization, stable ergonomics, and facilitated suturing.

Respondents showed mixed opinions about whether open surgery experience is a prerequisite for becoming an accomplished robotic surgeon. A large proportion (81.5%) viewed open experience as crucial to establish anatomical judgment and safe intraoperative decision-making. In contrast, a clear majority (66.4%) considered laparoscopic experience not indispensable for the acquisition of robotic competence. The need for pre-existing minimally invasive spatial awareness, familiarity with pneumoperitoneum, and experience in the management of restricted operative fields was underlined repeatedly in responses.

When assessing preoperative decision-making with regard to the choice of surgical approach, a number of key determinants have been reported by respondents. The majority of surgeons (59.7%) identify tumour characteristics, anatomic complexity, and need for vascular or biliary reconstruction. Other commonly cited factors included surgeon experience, institutional resources such as robotic platform availability, and patient-specific issues such as comorbidities or prior surgery. Fewer identified scheduling constraints or cost-related issues.

Surgeons were also asked to indicate their preferred operative approach in several clinical scenarios structured around the Tampa difficulty score. For non-anatomical resection of a hepatic tumour less than 3 cm located in segment III (TAMPA Group 1), respondents most frequently selected either a laparoscopic or robotic approach, with 64 (53.8%) and 53 (44.5%) choosing these options, respectively, while 2 (1.7%) favoured an open operation. When asked about a left lateral segmentectomy for malignant tumours larger than 3 cm (TAMPA Group 2), the distribution of responses shifted, with 78 (65.5%) opting for a robotic approach, 4 (3.4%) for an open approach, and 37 (31.1%) for a laparoscopic procedure. In the context of patients who had received neoadjuvant or adjuvant chemotherapy and should undergo right hepatectomy for a malignant tumour bigger than 3 cm (TAMPA Group 3), 49 (41.2%) respondents selected an open approach, while 58 (48.7%) opted for a robotic strategy and 12 (10.1%) for laparoscopy. For an extended right hepatectomy requiring lymphadenectomy and biliary reconstruction (TAMPA Group 4), respondents favoured an open approach (96; 80.7%), followed by robotic (20; 16.8%) and laparoscopic (3; 2.5%) options.

**Fig. 3:**
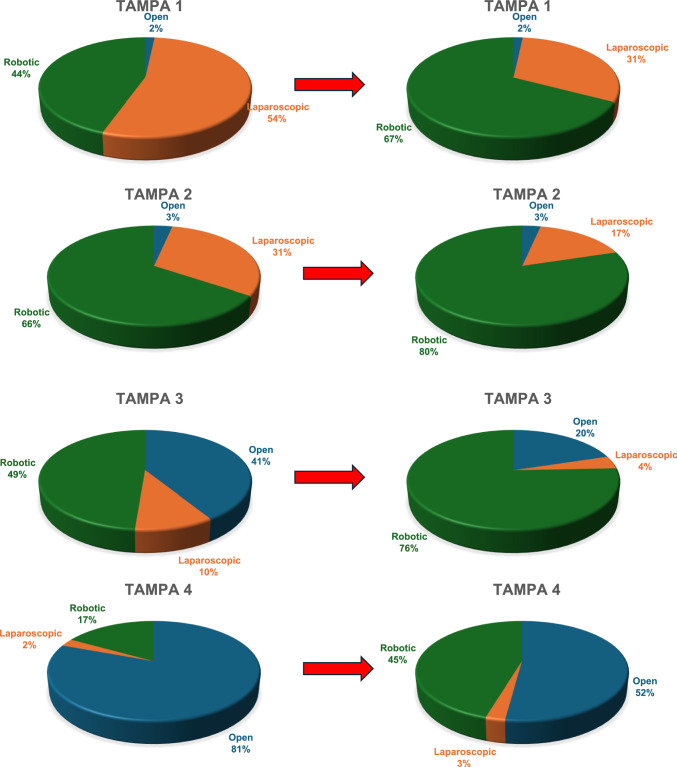
Surgeons’ preferences for open, laparoscopic, or robotic surgery in four predefined clinical scenarios based on the Tampa difficulty score. The left panel shows preferences according to the respondents’ current level of expertise, while the right panel shows preferences assuming equal technical proficiency across all surgical approaches. Results are expressed as percentages of respondents for each approach

These same clinical scenarios were reassessed under the assumption that surgeons possessed equal technical skill across open, laparoscopic, and robotic modalities. In the scenario of a non-anatomical resection of a small hepatic lesion in segment III, 37 (31.1%) selected the laparoscopic approach and 80 (67.2%) the robotic one, while 2 (1.7%) indicated a preference for open surgery. For a left lateral segmentectomy for malignant tumours larger than 3 cm, 95 (79.8%) preferred a robotic procedure, 4 (3.4%) an open operation, and 20 (16.8%) a laparoscopic approach. In cases of a right hepatectomy for a tumour bigger than 3 cm that underwent neoadjuvant or adjuvant therapy, 90 (75.6%) indicated a robotic preference, 24 (20.2%) an open preference, and 5 (4.2%) a laparoscopic choice. For procedures such as an extended right hepatectomy requiring lymphadenectomy and biliary reconstruction, 62 (52.1%) selected open surgery, 54 (45.4%) robotic surgery, and 3 (2.5%) laparoscopy. Figure 3 graphically represents the preferences expressed in the four proposed scenarios and how preferences change when skills are equal.

Surgeons were also asked about their preferred approach if a robotic procedure required conversion. In emergencies, the overwhelming majority indicated they would convert to an open approach (113; 95%), while a smaller proportion would convert to laparoscopy (6; 5%). A similar distribution was observed for conversions prompted by anesthesiological issues, with 107 (89.9%) selecting open conversion and 12 (10.1%) choosing a laparoscopic alternative. When asked about the preferred approach for emergency re-intervention following a robotic procedure, respondents again most commonly selected the laparoscopic approach (66; 55.5%), with smaller proportions opting for open (32; 26.9%) or robotic (21; 17.6%) re-intervention.

Finally, respondents were asked to consider the future of laparoscopy in HPB surgery. A majority indicated that laparoscopic HPB surgery would continue to exist alongside robotic surgery (72; 60.5%). Another group believed it would remain limited to selected procedures (30; 25.2%), while 17 (14.3%) anticipated that laparoscopic HPB surgery may disappear entirely.

## Discussion

This international survey offers a modern, application-oriented perspective on the use of robotic platforms in liver surgery, covering adoption rate, learning curve, choice of surgical technique based on procedure difficulties, safety issues in patient care, cost factors, and future directions. This targeted survey is aimed not at comparing which is better, but rather at documenting real-world perceptions and choice behavior of individuals actively engaged in robotic liver surgery today. The survey results contribute, thereby, to a growing, but still very diverse, worldwide perspective on robotic liver surgery.

The responses to the surveys have reaffirmed a marked increase in the availability of robotic platforms in HPB units over the last decade, although with a degree of variability. The da Vinci robotic platform has been identified as the most common robotic system available, although this is largely a result of the platform being available on the market for a longer period of time with a proven track record of use. Other, newer platforms such as Versius, Hugo RAS, as well as Hinotori, are still in the developmental stages with respect to liver surgery. This is consistent with recent surveys based on society, such as the E-AHPBA initiative, documenting high availability of platforms per responding center but great variability of procedural volumes and patient selections [4]. Noteworthy is that, according to this survey, the availability of robotic platforms is far from being a criterion for equal use. On the contrary, use is largely dependent on the center strategy, the surgical director, and the presence of experienced multidisciplinary staff.

Among the most evident signals that came out of the survey is the importance of structured training programs. The respondents generally viewed simulation-based training, proctoring, and dual-console mentoring as essential components of safe and sound implementation of robotic liver surgery. Such components are essential for a positive implementation experience, within which success is generally attributed to these components [10, 14–17]. Attending to the needs of the respondents, the learning curve for robotic liver surgery can be broken down from the point of competence to consolidation, culminating in the state of mastery, which is a multi-step learning curve that varies from one respondent to another with respect to the number of cases that need to be considered in overcoming these stages [18–21]. The importance of dual-console teaching in learning robotic surgery was extensively emphasized, especially in relation to tasks such as dissection, vascular control, and intracorporeal suturing [22–26].

The survey has shown a debate on the impact of previous laparoscopic skills on robotic liver surgery adoption. Even though a major part of the respondents have managed to emphasize the essential role of previous skills in open liver surgery, there have been beliefs that robotic systems can reduce some inherent drawbacks of laparoscopy, such as limited instrument dexterity. This attitude meant that a belief existed that even a limited number of experienced laparoscopists might be adept at minimally invasive liver surgery using a robotic system, provided that a full training program is available for them.

The survey responses also showed that the choice of approach is still basically dependent on tumor factors, anatomical, and reconstructive requirements. In the case of low-complexity procedures, namely anterolateral segment resections, there appeared to be no significant differentiations whether a laparoscopic, robotic, or open approach is used. In the case of posterosuperior, major hepatectomy, biliary, and vascular reconstructions, a robotic/open approach is clearly preferred.

The reasons cited for preferring robotic surgery were attributed to the advantage of robotics in difficult anatomical conditions, such as improved visualization, delicacy, and precision when working in a closed surgical environment. This correlates with experiences that reported improved access to regions VII–VIII and the caudate lobe, as well as easy completion of complicated suturing maneuvers [16, 27–34]. Lower rates of conversion for complicated rescissions using robotic surgery, as against laparoscopy, in high-volume institutions have been reported in several experiences referred to by respondents, with success largely dependent on the learning curve of the institution [9, 35, 36].

In particular, when patients were asked to have equal knowledge of all open, laparoscopic, and robotic methods, a preponderance of patients preferred the robotic method even in difficult cases. This indicates that factors such as availability, cost, etc., are significant contributors, but the limitation itself is not a reason to overlook the robotic method [4, 37].

Although the survey did not assess any objective perioperative outcome, ensuring patient safety as well as that of the team is considered a fundamental aspect when involving robotic systems in liver surgery. The availability of established perioperative procedures, experienced anesthesiologists, in addition to the presence of trained robotic staff, is paramount in ensuring that complications are averted. The respondents have a low threshold for conversion in cases involving uncontrolled bleeding, which is consistent with a safe approach involving the complexity of liver surgery. The institutions that are more organized in terms of workflow, high procedural volume, as well as a trained robotic team are considered better prepared for dealing with the challenges in the perioperative period, thus solidifying that the success in robotic liver surgery is highly dependent on organizational factors as opposed to technological prowess [38, 39].

Limitations of this research have a couple of drawbacks. Firstly, it is a survey, which is based on perceptions, not clinical outcomes, creating the potential for bias. Secondly, voluntary participation might have caused a bias toward already experienced individuals/entities when it came to robotic liver surgery. Thirdly, even though the research is worldwide, it might lack generalizability concerning developing countries. Fourthly, the number of experienced individuals might affect learning curves regarding the choice of the approach. Lastly, the research cannot identify a cause-and-effect relationship concerning a comparison of clinical outcomes that might occur when different approaches are used.

## Conclusion

This international survey gives a modern-day snapshot of practice habits, perspectives, and decision-making regarding robotic liver surgery. Key findings from this survey indicate a rising diffusion of robotic systems in hepatobiliary institutes with profound variability in the use, training models, and indications in robotic surgery. Even though robotic systems are on the rise, the adoption of these systems within liver surgery is highly dependent on the organizational structure, familiarity, and availability of training models.

Structured educational models, especially simulation-based training, proctoring, and two-console mentoring, appear to play a pivotal role in ensuring the safe implementation of robotic liver surgery. The learning curve has been perceived to be a multi-step process, which is dependent on procedural sophistication as well as existing surgical skills.

The choice of approach was mainly dependent on anatomical complexity. Although there is a tendency to consider laparoscopic and robotic approaches as similar alternatives for low-complexity resections, robotic, as well as open approaches, are considered preferred options for posterosuperior lesions, major hepatectomy, and complicated reconstruction. Crucially, assuming equal skills, several surgeons showed a bias toward robotic alternatives even in cases of complicated anatomy, implying that current, rather than inherent, challenges are still the dominant factors.

In conclusion, this survey substantiates the fact that future growth in robotic liver surgery is going to rely more on balancing inequality, standardizing training, organizational support, and economic viability, rather than pinning hopes on technological development alone. The results of this study serve as a basis for further collaboration with a common goal of standardizing robotic liver surgery practices globally.

## Data Availability

The data supporting the findings of this study are not publicly available due to institutional privacy restrictions and the confidential nature of the participating responses. An anonymized version of the dataset may be provided by the corresponding author upon reasonable request for academic and non-commercial purposes.
